# Case Hæmatemesis from Small Ulceration, Opening a Branch of the Gastric Artery.—Proving Fatal

**Published:** 1847-04

**Authors:** W. W. Reid

**Affiliations:** Rochester


ART. 111.—*Case Hæmatemesis from small ulceration, opening a branch of the gastric artery.—proving fatal.*—By W. W. Reid, M. D.

Dr. Flint.—The following case I send you, as one of much interest, and worthy of record in the Annals of Medicine.

A young woman, a chambermaid, aet. 25, for a year or more had had pain in the stomach; at times so severe as to disable her from her work for a few days. During the week previous to the 12th ult., she had more pain than she had had for some months, but was able to perform her usual labor, though she took some medicine from a Homœopathist. I should remark that she was very tender over the region of the stomach at all times, when she did not suffer acute pain; and her food would often produce uneasiness and distress in the stomach, yet she was not sick, and most of the time in the enjoyment of tolerable health.

On the evening of the 12th ult. she went to bed more unwell than usual, and before falling asleep, became sick at the stomach and vomited. In the morning of the 13th she arose and dressed herself, but was faint, and was obliged to go to bed. About this time she discovered that what she had thrown up in the night was blood, and which is reported to be about three pints, and nearly all blood. On the afternoon, she vomited again, a pint or more of clotted blood. On the 14th she threw up more. Then again on the 15th. From the 19th to the 22d, she was considered better and improving, tho' unable to be raised up without fainting. About noon on the 22d, she had something like a convulsion, (so the attendants say) became unconscious and somewhat comatose. She continued in this condition till the morning of the 23d, when she had another convulsion, and died during the afternoon of the 23d. She retched a great deal and threw up occasionally a small quantity of an inky black fluid.

Autopsy, 3 o'clock P. M., 7 hours after death. External appearance of the body emaciated, and very pallid. The chest and abdomen only were examined. The lungs generally of a dark gray color. The right one, superiorly and posteriorly, adherent, but otherwise perfectly sound.

The colon was very much contracted for more than half its length, but no appearance of disease. With these exceptions all the other viscera appeared healthy. The stomach was now removed and opened. It contained very little, and that chiefly mucus and water of an ash gray color. The right end, including half of the stomach, seemed contracted and thickened ; the mucous membrane especially, was very much corrugated, but no appearance of disease. The other half and large end, was thinner ; the mucous membrane without rugae, and three or four square inches of it was slightly injected, giving it a faint reddish hue, as if in a state of subacute inflammation. All the rest of the mucous and other tissues were free from disease. But on a close inspection, there was discovered, (and that almost by accident, it was so small,) near the edge of the aforesaid discolored portion, and almost three inches from the cardiac orifice, towards the great curvature of the stomach, a point, somewhat like a cicatrix, and in the center of it a depression, that resembled the Puncta Lacrymalis when large. The blunt end of a common silver probe readily entered it, and passed directly into a branch of the gastric artery, as it passed around behind the stomach. The probe being withdrawn, and introduced into the open end of the artery where it had been cut on removing the stomach, passed along some two inches under the serous membrane on the outer side, and then thro' the aforesaid orifice into the stomach. Immediately around this orifice, there were very slight appearances of former disease. The mucous membrane was smooth and seemed cicatrized—but the appearance was very slight, and did not cover an area greater than half a finger nail.

Thus the causes of the former pain and tenderness over the region of the stomach, and of the subsequent hæmorrhage and death, were fully and satisfactorily revealed. The case is interesting in several points of view. In the first place I can find but very few cases like it on record, where ulceration of the mucous membrane of the stomach penetrated an artery, even where ulceration was more extensive. Again, but a single cicatrix was discovered, and that so small, that it was, I might say, detected almost by accident; for the the rest of the mucous coat was healthy, and no one would suspect, or look for such a lesion in a membrane so generally sound. It may easily be imagined that similar cases may have often occurred, but have been over-looked in the examination. As Hæmorrhage from the stomach has always been obscure as to its aetiology, this case may serve to throw some light on this point, and perhaps account satisfactorily for more cases than at present are suspected.

Respectfully yours,

Rochester, March 4th, 1847. W. W. REID.

